# Role of isoenzyme M2 of pyruvate kinase in urothelial tumorigenesis

**DOI:** 10.18632/oncotarget.8114

**Published:** 2016-03-16

**Authors:** Haiping Zhou, Xing Wang, Lan Mo, Yan Liu, Feng He, Fenglin Zhang, Kuo-How Huang, Xue-Ru Wu

**Affiliations:** ^1^Department of Urology, New York University School of Medicine, New York, NY 10016, USA; ^2^Department of Pathology, New York University School of Medicine, New York, NY 10016, USA; ^3^Veterans Affairs New York Harbor Healthcare System Manhattan Campus, New York, NY 10010, USA

**Keywords:** bladder cancer, tumorigenesis, precancerous, PKM2, urothelium

## Abstract

The conversion of precancerous lesions to full-fledged cancers requires the affected cells to surpass certain rate-limiting steps. We recently showed that activation of HRAS proto-oncogene in urothelial cells of transgenic mice causes simple urothelial hyperplasia (SUH) which is persistent and whose transition to low-grade papillary urothelial carcinoma (UC) must undergo nodular urothelial hyperplasia (NUH). We hypothesized that NUH, which has acquired fibrovascular cores, plays critical roles in mesenchymal-to-epithelial signaling, breaching the barriers of urothelial tumor initiation. Using proteomics involving two-dimensional gel electrophoresis, immunoblotting with pan-phosphotyrosine antibody and MALDI-mass spectrometry, we identified isoform 2 of pyruvate kinase (PKM2) as the major tyrosine-phosphorylated protein switched on during NUH. We extended this finding using specimens from transgenic mice, human UC and UC cell lines, establishing that PKM2, but not its spliced variant PKM1, was over-expressed in low-grade and, more prominently, high-grade UC. In muscle-invasive UC, PKM2 was co-localized with cytokeratins 5 and 14, UC progenitor markers. Specific inhibition of PKM2 by siRNA or shRNA suppressed UC cell proliferation via increased apoptosis, autophagy and unfolded protein response. These results strongly suggest that PKM2 plays an important role in the genesis of low-grade non-invasive and high-grade invasive urothelial carcinomas.

## INTRODUCTION

Tumorigenesis of the epithelial tissues is marked by multiple steps from normal-appearing architectures to precancerous lesions and to full-fledged neoplasms [1]. Each of these steps is driven by accumulative alterations on genomic, genetic and epigenetic levels. Urothelial tumorigenesis appears to follow a similar path [2–4], although exactly what drives a particular step within a specific phenotypic pathway remains poorly understood.

Most urothelial carcinomas (UC) in humans fall into two major phenotypic categories: low-grade papillary UC and high-grade invasive UC [2–6]. The former accounts for about 70% of all UC, is commonly multifocal and recurs frequently after surgical resection, but exhibits limited potential to progress to the invasive stage. Histopathological studies showed that simple and papillary urothelial hyperplasias often coexist with or precede this UC variant, thus being the likely precursor lesions [7]. The high-grade invasive UC accounts for 25-30% of all UC and is highly prone to progression to local and distant metastases [8]. It is presently unclear what molecular event(s) must take place in order for the precursor lesion within each of the two major phenotypic pathways to become full-fledged UC.

An effective approach to dissect the dynamic process of tumor formation is the use of genetically engineered mice. By expressing a constitutively activated HRAS in transgenic mouse urothelia under the control of a mouse uroplakin II promoter, we previously observed that HRAS-mediated urothelial tumorigenesis proceeds in a time- and transgene-dosage-dependent manner [9, 10]. In young heterozygous *Upk2*-HRAS^*/WT^ transgenic mice, urothelium undergoes simple hyperplasia which persists for about 10 months without reversion to normal urothelium or transition to full-fledged carcinoma [9], suggesting a rate limiting step that prevents urothelial tumor initiation. Between 11-18 months, the heterozygous *Upk2*-HRAS^*/WT^ transgenic mice develop nodular hyperplasia with a distinguishing feature in the appearance of multiple fibrovascular cores. From 19-28 months, approximately 60% of the heterozygous *Upk2*-HRAS^*/WT^ mice develop low-grade, papillary UC that bears strong resemblance to the human counterpart [9]. Such a sequence of tumorigenesis (i.e., simple hyperplasia to nodular hyperplasia to low-grade papillary UC) can be reproduced in homozygous *Upk2*-HRAS^*/*^ transgenic mice, albeit in a much shortened time course [10]. Thus, simple hyperplasia, nodular hyperplasia and low-grade papillary UC occur at birth, 1 month and 3 months of age, respectively. It appears from both the heterozygous and the homozygous *Upk2*-HRAS transgenic mice that the emergence of the fibrovascular cores in the nodular hyperplasia is critical to providing mesenchymal-to-epithelial communication, allowing growth factors from the bloodstream and/or mesenchymal cells to over-activate the mitogenic and survival pathways in urothelial cells and break down the barriers to urothelial tumor initiation [10].

Tumor cells are fundamentally different from their normal counterparts in how glucose is metabolized. Instead of the energy-efficient mitochondrial oxidative phosphorylation in normal cells, glucose undergoes energy-inefficient glycolysis in tumor cells even in the presence of sufficient oxygen supply, hence the term “aerobic glycolysis” or “Warburg effect” [11, 12]. Pyruvate kinase (PK) is a key enzyme that catalyzes the final, rate-limiting step of glycolysis by transferring a phosphate group from phosphoenolpyruvate to ADP [13, 14]. The muscle-type PK (PKM) gene is ubiquitously expressed and alternatively spliced giving rise to two mRNA products that differ in the mutually exclusive use of exon 9 (PKM1) or exon 10 (PKM2) [15]. The general consensus is that PKM1 is expressed in normal adult issues, whereas PKM2 is expressed primarily during embryogenesis [11–13]. Interestingly, in fast-proliferating and tumor cells, the PKM isoform can be switched from PKM1 to PKM2, a switch associated with a reduced pyruvate kinase activity [11–13]. The result is an increased accumulation of glycolytic intermediates which are shunted toward the pentose phosphate and hexosamine pathways, thus favoring the synthesis of biomass building blocks such as amino acids, lipids and nucleic acids [15, 16]. PKM2 activity is also subject to allosteric regulation by tyrosine phosphorylation or by binding to phosphotyrosine-containing peptides [15, 17].

To begin to define the molecular driver(s) underlying the transition between urothelial simple hyperplasia and nodular hyperplasia, we have conducted a series of molecular profiling studies, one of which aimed to identify proteins that act in the receptor tyrosine kinase (RTK) pathway whose activation often replies on tyrosine phosphorylation. Here we report the identification of isoform 2 of pyruvate kinase (PKM2) as the principal tyrosine-phosphorylated protein that was upregulated during nodular hyperplasia formation in *Upk2*-HRAS transgenic mice. Our data suggest that PKM2 plays a critically important role in the genesis of urothelial carcinomas.

## RESULTS

### Identification of PKM2 as the major tyrosine-phosphorylated protein during the formation of nodular urothelial hyperplasia and papillary urothelial carcinoma

**Figure 1:**
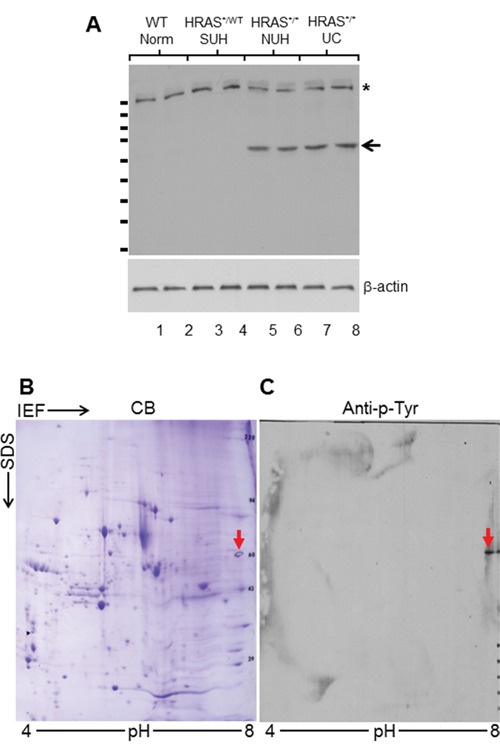
Proteomic identification of isoform M2 of pyruvate kinase (PKM2) in HRAS-induced, low-grade papillary urothelial carcinoma in transgenic mice **A.** Western blotting of total protein extracts (two mice per phenotype) from 3-month old wild-type (WT) mice exhibiting normal urothelium (Norm); 3-month old heterozygous *Upk2*-HRAS^*/WT^ transgenic mice exhibiting simple hyperplasia (SUH); 1-month old homozygous *Upk2*-HRAS^*/*^ transgenic mice exhibiting nodular urothelial hyperplasia (NUH) containing fibrovascular cores; and 5-month old homozygous *Upk2*-HRAS^*/*^ transgenic mice exhibiting low-grade papillary urothelial carcinomas (UC). An anti-phosphotyrosine antibody detected a 180-kDa species in all samples (asterisk) and a 60-kDa species only in nodular urothelial hyperplasia and low-grade papillary UC (arrow). Anti-β-actin was used as a loading control (lower panel). Short, horizontal bars on the left of the top panel denote molecular weight standards (top to bottom: 170, 130, 100, 72, 55, 40, 33, 24, and 17-kDa). **B.** Two-dimensional gel electrophoresis (isoelectric focusing or IEF (ampholyte pH range: 4-8) in the first dimension and SDS-PAGE in the second dimension), followed by Coomassie blue (CB) staining of total protein extracts of UC (as shown in panel A). Multiple protein spots were revealed. **C.** A replica of panel B was transferred to Immobilon-PVDF and immuno-blotted with anti-phosphotyrosine (anti-p-Tyr) detecting a single spot with a molecular mass of 60-kDa that corresponded to a spot in CB-stained SDS-PAGE. The spot was then excised for tryptic digestion, HPLC and mass spectrometry (see Methods and Results).

To identify growth-promoting signals that were activated via tyrosine phosphorylation along the receptor-tyrosine-kinase (RTK) pathway thus potentially overcoming the rate-limit step of urothelial carcinoma initiation, we first employed one-dimensional (1-D) SDS-PAGE followed by Western blotting using an antibody against pan-phosphotyrosine capable of reacting with all tyrosine-phosphorylated proteins. We utilized, as our starting materials, (i) normal urothelia (NU) from wild-type mice, (ii) simple urothelial hyperplasia (SUH) from heterozygous *Upk2*-HRAS^*/WT^ transgenic mice (2-3 months old; [9]), (iii) nodular urothelial hyperplasia (NUH) from young homozygous *Upk2*-HRAS^*/*^ transgenic mice (1-month old; [10]), and (iv) low-grade, papillary urothelial carcinoma (UC) from older homozygous *Upk2*-HRAS^*/*^ transgenic mice (5-months old; [10]) (Figure 1A). The major protein species that contained the phosphotyrosine were surprisingly few, i.e., only two major bands with molecular weights of around 180-kDa (Figure 1A; asterisk) and 60-kDa (Figure 1A; arrow). Importantly, the 60-kDa phosphotyrosine-containing protein species was present in NUH and low-grade papillary UC, but not in NU or SUH (Figure 1A; arrow), making it an interesting target for further analysis. We next performed 2-D gel electrophoresis (Figure 1B) and, upon Western blotting, a well-defined spot with a molecular weight of 60-kDa and a PI of about 7.0 was reactive to the phosphotyrosine antibody (Figure 1C). The matching spot on a duplicate, Coomassie blue-stained gel was subject to in-gel trypsin digestion, and the resultant tryptic digests were subject to MALDI-mass spectrometry (see Methods for details).

From mass spectrometry, a total of 12 peptides matched the exon sequences of pyruvate kinase gene in areas shared by isoforms M1 (PKM1) and M2 (PKM2), with a 97.9% total amino acid sequence coverage. Two additional peptides matched only to exon 10 that encodes the specific sequence for PKM2 of the pyruvate kinase gene, whereas none of the peptides matched only to exon 9 (encoding PKM1) of the gene.

### Overexpression of PKM2 in low-grade as well as high-grade mouse and human UC and UC cell lines

**Figure 2:**
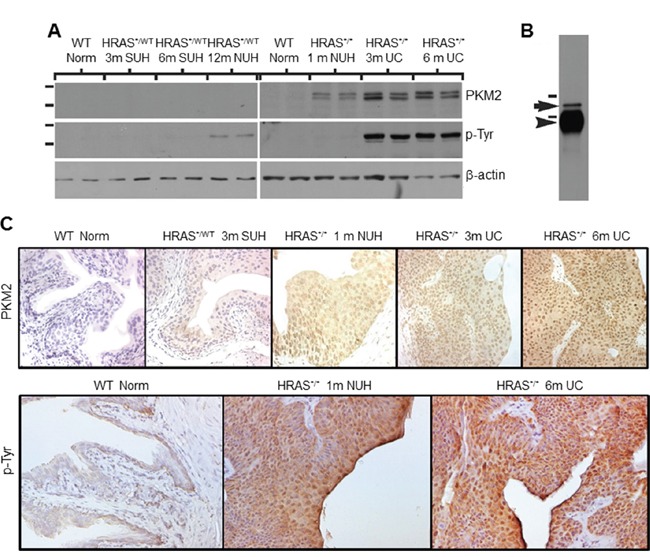
Expression levels and subcellular localization of PKM2 in low-grade urothelial lesions induced by activated HRAS **A.** Western blotting of total protein extracts of normal urothelial cells from wild-type mice (6 months of age), simple urothelial hyperplasia (SUH) from 3- and 6-month-old heterozygous *Upk2*-HRAS^*/WT^ transgenic mice (abbreviated as HRAS^*/WT^), nodular urothelial hyperplasia (NUH) from 12-month-old heterozygous *Upk2*-HRAS^*/WT^ transgenic mice and 1-month-old homozygous *Upk2*-HRAS^*/*^ transgenic mice, and low-grade papillary urothelial carcinoma (UC) from 3- and 6-month-old homozygous *Upk2*-HRAS^*/*^ transgenic mice, using anti-PKM2, anti-phosphotyrosine and anti-actin antibodies. Note the first appearance of PKM2 and phosphorylated PKM2 in nodular urothelial hyperplasia followed by an increased intensity in low-grade UC. The doublet of PKM2 most likely represented the non-phosphorylated and phosphorylated versions of PKM2. **B.** Immunoprecipitation (IP) using as starting materials total proteins from low-grade papillary UC (as shown in (A), last two lanes), using anti-phosphotyrosine antibody followed by Western blotting using anti-PKM2 antibody. Note the specific identification of the 60-kDa PKM2 (arrow). Arrowhead denotes the IgG heavy chain present in the IP product reactive with the secondary antibody during Western blotting. Short horizontal bars denote two of the molecular weight standards (top, 72-kDa; bottom, 55-kDa). **C.** Immunohistochemistry using anti-PKM2 and anti-phosphotyrosine showed that the expression of PKM2 and p-PKM2 corresponded well with that of the Western blotting and that prominent nuclear staining was present. Magnification of panels in C: 200x.

To validate our proteomic data and to assess whether the expression of PKM2 per se, not just its tyrosine-phosphorylated version, was upregulated in different variants of UC, we carried out Western blotting and immunohistochemical staining using tumor specimens from transgenic and knockout mice, human UC and human UC cell lines. In our *Upk2*-HRAS transgenic mice that mimic the low-grade papillary UC pathway [10], PKM2 was undetectable in normal urothelia from wild-type littermates or in SUH of the young heterozygous *Upk2*-HRAS^*/WT^ mice (2-10 months of age) (Figure 2A and 2C). PKM2 started to appear during NUH of the old heterozygous *Upk2*-HRAS^*/WT^ mice (>10 months) and the young homozygous *Upk2*-HRAS^*/*^ mice (1 month), and its high expression continued in low-grade papillary UC (Figure 2A and 2C). The timing of tyrosine phosphorylation of PKM2 in general corresponded with PKM2 overexpression (Figure 2A), as also evidenced by immunoprecipitation with anti-phosphotyrosine antibody followed by Western blotting with anti-PKM2 antibody (Figure 2B). Nuclear localization of both PKM2 and phosphorylated PKM2 was evident in NUH and low-grade papillary UC (Figure 2C).

**Figure 3:**
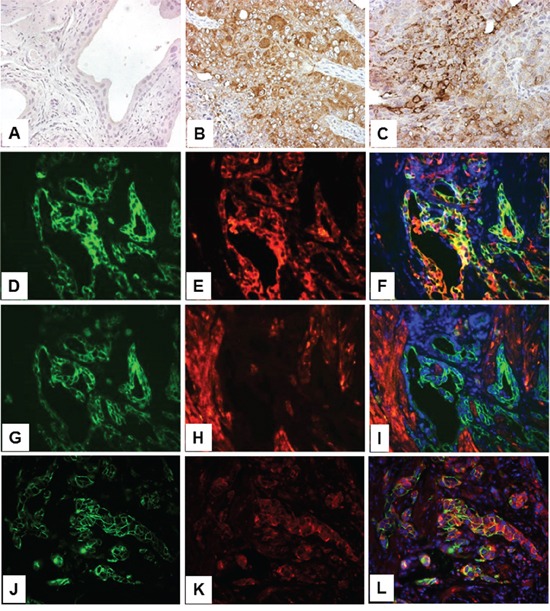
Expression of PKM2 in high-grade, muscle-invasive urothelial carcinoma of the transgenic mice Normal mouse bladder (A) and those from transgenic mice expressing HRAS and lacking p53 in urothelium (*Upk2*-HRAS^*/WT^/^Upk2^-cre/p53^lox/lox^) that developed muscle-invasive urothelial carcinoma (B and C) were subject to anti-PKM2 immunohistochemical staining **A-C.** Double immunofluorescence staining was also performed on invasive UC lesions using anti-cytokeratin 5 (K5, green; panel **D.**) and anti-PKM2 (red; panel **E.**) with their overlay shown in **F.** An adjacent section of (D) was double-stained using anti-K5 (panel **G**) and anti-PKM1 (panel **H**) with their overlay shown in **I.** Finally, double labeling was also performed using anti-K14 (panel **J**) and anti-phosphor-PKM2 (Y105) (panel **K**) with overlay shown in **L.** Note that normal urothelium lacks PKM2 expression and invasive urothelial cells strongly express PKM2; and that, while PKM2- and phosphor-PKM2-positive cells express K5 and K14, those that express PKM1 do not express K5. All panels: 200x.

In our compound transgenic mice expressing HRAS and lacking p53 (*Upk2*-HRAS^*/WT^/*Upk2*-cre/p53^lox/lox^) that mimic the high-grade, muscle invasive UC pathway [18], we observed marked upregulation of PKM2 in invasive UC cells (Figure 3B and 3C). Additionally, PKM2 was strongly expressed in cells positively labeled for cytokeratins 5 and 14 (Figure 3D-3F; Figure 3J-3L), progenitor cell markers of invasive UC [19, 20], whereas PKM1, stained in adjacent sections, was expressed primarily in cells negative for cytokeratin 5 (Figure 3G-3I). Nuclear PKM2 in invasive UC was not as prominent as in non-invasive UC, suggesting a genetic alteration-specific and/or UC variant-specific event.

**Figure 4:**
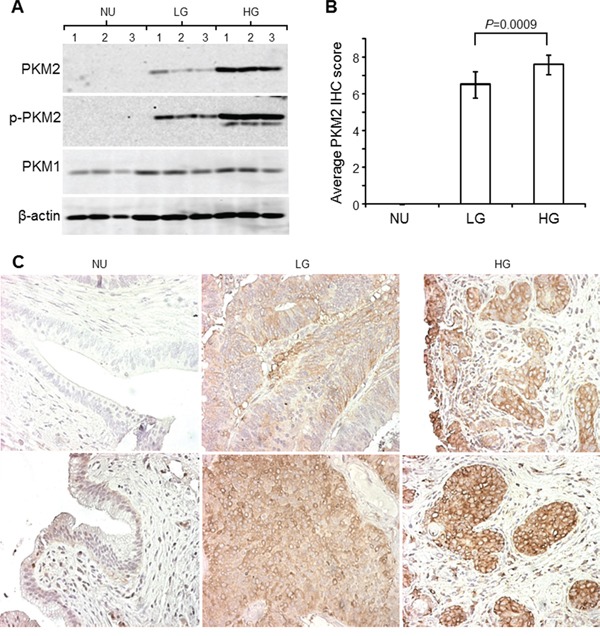
Expression of PKM2 in human urothelial carcinoma **A.** Total proteins extracted from freshly dissected normal urothelia (NU), low-grade (LG) non-invasive UC and high-grade (HG) invasive UC (three cases/group) were resolved by SDS-PAGE, electrotransferred to Immobilon-PVDF and immune-blotted with antibodies against PKM2, phosphorylated PKM2 (Y105), PKM1 and β-actin. **B** and **C.** The aforementioned three groups of specimens (N=6, 10 and 10 for groups NU, LG and HG, respectively) were paraffin-embedded, sectioned and immunohistochemically stained with anti-PKM2. The staining intensity and area were scored (see Methods for details), with the results tabulated in (B) and representative images shown in (C). Note the lack of expression, moderate expression and strong expression of PKM2 and phosphorylated PKM2 (assessed by Western blotting) in normal urothelia, low-grade UC and high-grade UC, respectively. Also note the only slightly increased expression of PKM1 in urothelial carcinoma compared to normal urothelia (A) and high-grade UC. Magnification for all panels in (C): 200x.

The human UC cohort that we used for the preliminary survey comprised 6 cases of normal urothelia from patients undergoing prostatectomy, 10 cases of low-grade papillary UC obtained from biopsies during transurethral UC resection and 10 cases of high-grade, muscle invasive UC from patients undergoing radical cystectomy. PKM2 was absent (Figure 4A; Figure 4C, upper left) or present in very low amount (Figure 4C, lower left) from normal urothelia and strongly expressed in low-grade papillary UC (Figure 4A; Figure 4C, middle panels) and more so in high-grade invasive UC (Figure 4A; Figure 4C, right panels) by both Western blotting (Figure 4A) and immunohistochemistry (Figure 4B and 4C). The same trend held for phosphorylated PKM2 (Y105) (Figure 4A). PKM1 expression, on the hand, did not show significant difference among the three groups compared (Figure 4A; data not shown).

**Figure 5:**
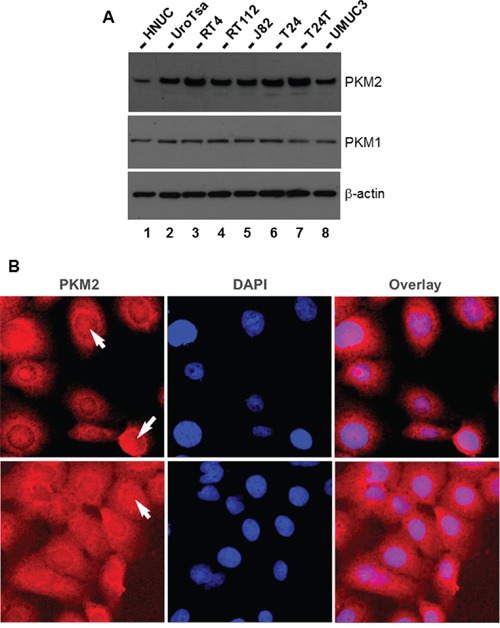
Expression and localization of PKM2 in cultured human urothelial carcinoma cell lines **A.** Western blotting of total protein extracts from primary cultured human normal urothelial cells (HNUC), SV40T-immortalized urothelial cells (UROtsa), cell lines derived from low (RT4) and moderate (RT112) grade human UC, and cell lines derived from high-grade human UC (J82, T24, T24T (a metastatic derivative of T24), and UMUC3). Note that, with the exception of UMUC3, cell lines derived from both low- and high-grade human UC overexpress PKM2, but not PKM1, compared with primary cultured and immortalized human urothelial cells. **B.** Immunofluorescence staining of T24 cells with DAPI counterstaining showing both cytoplasmic and nuclear localization of PKM2. Magnification: 400x.

To set a stage for the necessary *in vitro* studies, we surveyed several established cell lines initially derived from and representing different grades and stages of human UC [21]. We found that, whereas PKM1 was relatively uniformly expressed in all the cell lines examined, PKM2 was more highly expressed in UC-derived cell lines and SV40T oncogene-immortalized human urothelial cells than primary-cultured human normal urothelial cells (HNUC) (Figure 5A). The only exception was the UMUC3 cell line, which expressed less PKM2 than other UC cell lines. Immunofluorescent staining of T24 cells revealed both cytoplasmic and nuclear staining of PKM2 (Figure 5B).

### Down-regulation of PKM2, but not PKM1, led to significantly reduced UC cell Proliferation

**Figure 6:**
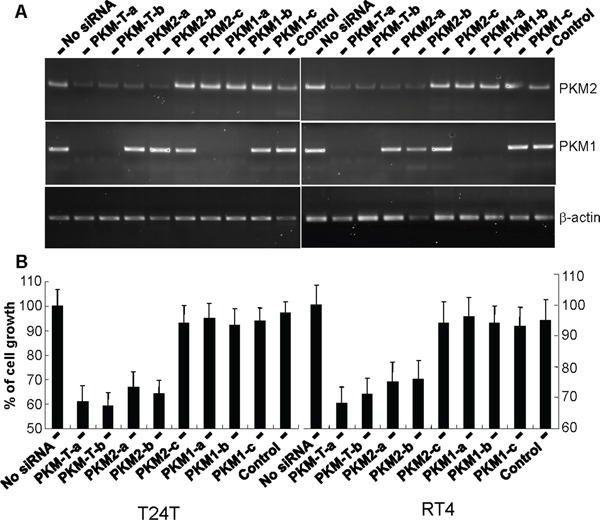
Inhibition of urothelial carcinoma cell proliferation via down-regulation of PKM2 but not PKM1 Bladder cancer cell lines T24T and RT4 were transiently transfected with siRNAs designed for the common mRNA regions shared by PKM2 and PKM1 (two independent siRNAs) or specific for PKM2 or PKM1 (three independent siRNAs per isoform). The expression of PMK2 and PKM1 expression was assessed by RT-PCR using isoform-specific primers and the cell proliferation status was determined with and without siRNA knockdown using the WST-1 assay. Note, in both cell lines, the intact PKM2 and PKM1 in cells without siRNA transfection or with transfection of siRNA of EGFP (control), the knockdown of both PKM2 and PKM1 in cells with siRNA for the common regions, the specific knockdown of PKM2 in cells with siRNA for PKM2, and the specific knockdown of PKM1 in cells with siRNA for PKM1. The only two exceptions were one each of the siRNAs for PKM2 (PKM2-c) and PKM1 (PMK1-c) that had low efficiency score on siRNA design software and did not work well. Also note that the knockdown of both PKM2 and PKM1 and the knockdown of PKM2 alone, but not knockdown of PKM1 alone, led to significantly reduced cell proliferation. Finally, the third siRNA designed for PKM2 (PKM2-c) that did not work well had little effect on cell proliferation.

To begin to determine the effects of PKM2 overexpression on UC cell growth, we performed knockdown experiments initially using siRNAs corresponding to the common regions of PKM2 and PKM1 and siRNAs specific for PKM2 (corresponding to exon 10 sequence) and those for PKM1 (corresponding to exon 9). Transient transfection of T24T and RT4 human UC cell lines followed by RT-PCR established the markedly reduced expression of both PKM2 and PKM1 by siRNAs corresponding to common regions (PKM-T-a and PKM-T-b); reduced expression of PKM2 by 2 of the 3 exon 10-specific siRNAs (PKM2-a and PKM2-b); and reduced expression of PKM1 by 2 of the three exon 9-specific siRNAs (PKM1-a and PKM1-b) (Figure 6A). The third siRNA for PKM2 (PKM2-c) and that for PKM1 (PKM1-c), which had relatively lower efficiency scores (2.5) by siRNA design software, did not perform as well as the other siRNAs (3.5 to 5). Overall, knockdown results were highly consistent with the two independent cell lines (Figure 6B). After 24 hours of incubation, the cell proliferation status was quantified for siRNA-transfected cells using WST-1 assay. It was evident that inhibition of PKM2 or both PKM2 and PKM1, but not PKM1 alone, had significantly reduced cell proliferation. Consistent with this overall result, the lack of PKM2 down-regulation by the poor-performing, third PKM2 siRNA did not lead to inhibition of cell proliferation (Figure 6B).

### Down-regulation of PKM2 resulted in increased UC apoptosis, autophagy and unfolded protein response

**Figure 7:**
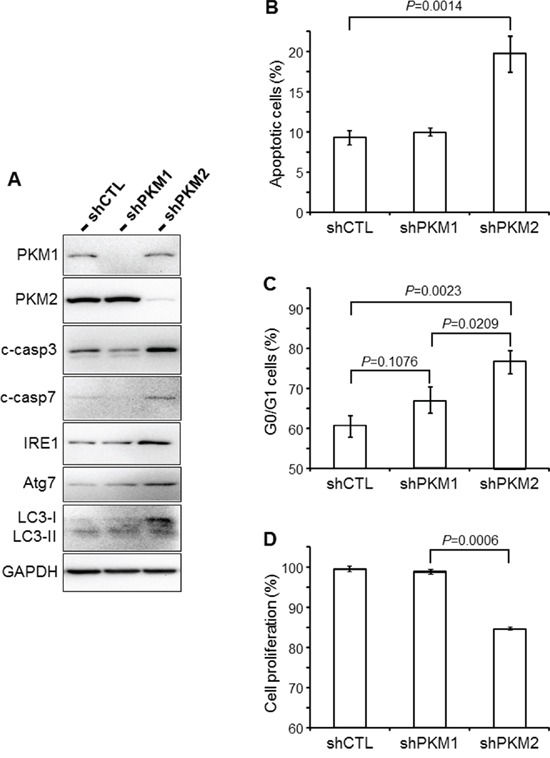
Cellular effects of PKM2 knockdown **A.** UC cell line T24 was infected with retroviral control vector only (shCTL) or that containing shRNA specific for PKM1 (shPKM1) or shRNA specific for PKM2 (shPKM2). Thirty-six hours after infection, the cells were lysed and total proteins subjected to Western blotting using antibodies against PKM1, PKM2, cleaved caspases 3 and 7 (c-casp3 and c-casp7), IRE1, Atg7, LC3-I/II and GAPDH. Note that the down-regulation of PKM2, but not PKM1, led to increased levels of markers indicative of apoptosis, autophagy and unfolded protein response. **B** and **C.** Fluorescence-activated cell sorting (FACS) of annexin V-stained T24 cells (B), or of propidium iodide-stained T24 cells (C) that had been infected with shRNA-containing viral vectors as indicated. **D.** WST-1 cell proliferation assay of T24 cells infected with shRNA-containing viral vectors as indicated. Note that the PKM2 knockdown significantly increased apoptosis (B) and G0/G1 distribution (C) and significantly decreased the proliferation (D) of T24 cells.

To further study the effects of down-regulating PKM2 in UC cells, we transduced T24 cells with lentiviruses containing shRNAs specific for PKM1 or PKM2. A significant and specific knockdown was achieved as evidenced by Western blotting using an anti-PKM1 or anti-PKM2 antibody (Figure 7A). Western blotting also showed significantly increased levels in PKM2-knockdown cells of apoptotic markers cleaved caspase 3 and 7, marker for unfolded protein response IRE1 and markers for autophagy Atg7 and LC3-I/II. These changes corresponded well with increased percentage of apoptotic cells (Figure 7B) and cells in the G0/G1 phase (Figure 7C) by fluorescence-activated cell sorting and decreased cell proliferation (Figure 7D).

## DISCUSSION

Of all the genetic alterations that have been found in human low-grade papillary UC, those that activate the receptor tyrosine kinase (RTK)/RAS/PI3K pathway are by far the most prevalent (e.g., 45-75% of the cases with FGFR3 mutations; 10-20% with RAS mutations; 25% with PI3K mutations; 6-8% with PTEN mutations; 5-8% with RAF mutations) [2–4]. This, plus the observation that most of these mutations are non-overlapping in a given UC and that several genes such as FGFR3 and RAS are overexpressed in the absence of mutation, suggests that the RTK/RAS/PI3K pathway is activated in most, if not all, of the low-grade papillary UC. Despite the exceedingly high frequency, whether the activation of this pathway by itself is tumorigenic remains controversial [22]. In fact, experimental evidence from genetically engineered mice argues, in large part, against the activation of this pathway as the driver for low-grade papillary UC. For instance, urothelium-specific expression of an FGFR3 mutant (K644E) that constitutively activated the kinase domain of FGFR3 in transgenic mice up to 15 months of age failed to elicit any urothelial abnormality [23]. Expressing an HRAS mutant (G12V) from its endogenous promoter in all tissues including the urothelium via a knock-in approach also did not produce any discernible phenotypic changes over a year period [24]. Similarly, mice with PTEN deletion in the urothelium by two independent groups developed primarily urothelial hyperplasia, with only 10-20% of the mice forming tumors after 12-18 months [25, 26]. The low penetrance and long latency for some activated components and the complete lack of tumorigenesis for others in the RTK/RAS/PI3K pathway strongly suggest that the urothelial cells are relatively refractory to the oncogenic assaults solely from this pathway. They also imply that certain cooperative events are required in order for these cells to be fully transformed and tumors to develop.

As shown in our present work, one such event that could collaborate with the activated RTK/RAS/PI3K pathway to accelerate urothelial tumorigenesis is the overexpression of PKM2. This conclusion is based on our findings that PKM2 is the major tyrosine-phosphorylated protein upon low-grade papillary UC formation (Figure 1A); that it is switched on during nodular urothelial hyperplasia, an immediate precursor of low-grade papillary UC, but not in normal urothelium or simple urothelial hyperplasia (Figures 1A and 2A); that it is consistently expressed in mouse and human low-grade papillary UC and human UC cell lines derived from low-grade papillary UC (Figures 1–5); and that specific down-regulation of PKM2, but not its spliced isoform PKM1, inhibits UC cell proliferation (Figures 6 and 7) by suppressing cell-cycle progression and inducing apoptosis, autophagy and unfolded protein response (Figure 7). Not only does PKM2 appear to play an important role in low-grade papillary UC formation, it may also contribute to invasive urothelial tumorigenesis based on the data we presented here. Thus, the upregulation of PKM2 is prominent in muscle-invasive lesions of both mouse models and human patients (Figures 3 and 4). Additionally, knockdown of PKM2 reduces the proliferation of UC cell lines (T24 and T24T) derived from high-grade invasive UCs (Figures 6 and 7). Collectively, our data establishing the overexpression of PKM2 and their effects on UC growth of both phenotypic pathways of UC provide an example whereby epigenetic alterations can collaborate with genetic alterations to promote urothelial tumor formation. This finding is particularly significant in light of the fact that some of the genetic alterations such as the loss of both p16Ink4a and p19Arf, events prevalent in human UC, actually failed to collaborate with activated HRAS to initiate urothelial tumors [10].

While the mechanism by which PKM2 is upregulated during urothelial tumorigenesis in our *Upk2*-HRAS transgenic mice is beyond the scope of the present study, it is unlikely to be due to RAS activation alone. This is because the activation of HRAS in young animals of the same transgenic line that exhibits simple urothelial hyperplasia did not induce PKM2 (Figures 1 and 2). The fact that PKM2 overexpression coincides with the occurrence of the fibrovascular cores during the nodular hyperplastic stage suggests that growth factors from the mesenchymal cells and/or the bloodstream are key to the upregulation of PKM2. As we noted previously, mesenchymal-to-urothelial signaling may be critical for over-activating AKT and STAT3/5 and functionally disabling PTEN [10]. Some of these events have already been shown to upregulate PKM2 in non-urothelial cells [27]. It remains to be seen as to whether inhibiting some of these upstream growth promoters can downregulate PKM2 and in turn prevent urothelial tumor formation and recurrence.

Additional studies are also required to determine exactly how PKM2 overexpression leads to urothelial tumor formation, although there is likely a parallel to the growth-promoting effects of PKM2 in non-urothelial cells. For instance, switching from PKM1 to PKM2 in urothelial cells (Figures 3 and 4) and further reduction of the enzymatic activity of PKM2 by tyrosine phosphorylation (Figures 1–4) could reduce glycolysis and divert glycolytic intermediates for anabolic processes in favor of urothelial proliferation [11–13]. Growth factors released from the fibrovascular cores could complex with cytoplasmic β-catenin in the urothelial cells and together translocate into the nuclei to transcribe cell-cycle regulators and accelerate cell-cycle transition [28]. PKM2 may also work in concert with hypoxic factors such as HIF to fuel urothelial proliferation when oxygen is high demand and short supply [27]. Additionally, the antagonistic effects of PKM2 to apoptosis, autophagy and unfolded protein response (Figure 7) could enhance cell survival, further bolstering urothelial proliferation. Finally, as for the high-grade muscle-invasive UC, PKM2 is strongly expressed in the progenitor cells that express cytokeratins 5 and 14 (Figure 3), suggesting a potential role of PKM2 in the renewal, fate determination and amplification of the progenitor cells for the muscle-invasive UC [18–20].

Beyond its role in urothelial tumorigenesis, our finding on PKM2 may have practical implications in aiding the diagnosis and treatment of UC. Thus far, few biomarkers exist to reliably predict urothelial tumor progression along the two major phenotypic pathways [29–31]. Even fewer markers have been investigated to predict the transition from the precancerous lesions (urothelial hyperplasia in the low-grade papillary UC pathway; and CIS in the high-grade muscle-invasive UC pathway) to the full-blown UC. A follow-up study involving a large UC patient cohort encompassing all the stages and grades of UC with clinical outcome information is needed to address some of these questions. Another important challenge in managing UC is the natural and adaptive resistance to conventional chemotherapeutics, such as cisplatin and related drugs via, frequently, the evasion of apoptotic and autophagy responses [32]. It is worth noting that the down-regulation of PKM2 by siRNA or shRNA strongly induced these activities in the UC cell lines we tested (Figures 6 and 7). PKM2 inhibition may therefore represent an unconventional way through a complete different mechanism than the conventional DNA-alkylating agents to treat UC. It will be of considerable interest to find out whether PKM2 inhibition and chemotherapeutics are additive or even synergistic in therapeutically inhibiting advanced UC.

## MATERIALS AND METHODS

### Genetically engineered mice and their urothelial lesions

Transgenic mice bearing a constitutively active HRAS (Q61L) oncogene under the control of a 3.6-kB mouse uroplakin II promoter (*Upk2*-HRAS) developed urothelial lesions in time- and transgene-dosage-dependent manners. The heterozygous (*Upk2*-HRAS^*/WT^) mice exhibited simple urothelial hyperplasia between 2-10 months of age, 60% of which evolved into low-grade papillary UC between 11-28 months of age [9]. The homozygous mice (*Upk2*-HRAS^*/*^) developed simple urothelial hyperplasia at birth, nodular urothelial hyperplasia at 1 month and low-grade papillary UC between 3-6 months before all succumbed to tumor-caused urinary outlet obstruction and renal failure [10]. Age- and gender-matched wild-type (WT) mice harboring normal urothelia were used for comparative purposes in all applicable experiments and all mice were kept in an inbred FVB/N background. Another series of the mice used were compound mice expressing one allele of activated HRAS (*Upk2*-HRAS^*/WT^) and lacking both alleles of p53 (*Upk2*-cre/p53^lox/lox^) [18]. These mice first developed carcinoma in situ (3-7 months of age) and then high-grade muscle-invasive UC (8-12 months). All mouse studies were carried out after the official approval of a study protocol by the Institutional Animal Care and Use Committee (IACUC) of New York University School of Medicine.

### Proteomic identification of tyrosine-phosphorylated proteins during urothelial tumorigenesis

Total proteins (60 μg/lane) from normal urothelia of the WT mice, simple urothelial hyperplasia of the *Upk2*-HRAS^*/WT^ (3 months of age), nodular urothelial hyperplasia of the *Upk2*-HRAS^*/*^ mice (1 month of age) and low-grade papillary UC of the *Upk2*-HRAS^*/*^ (5 months of age) were dissolved in sample loading buffer containing 2% SDS, 50 mM Tris/HCl (pH 7.4), 150 mM NaCl and 5% β-mercaptoethanol and boiled for 5 min. The soluble proteins were resolved on a 15% SDS-PAGE, transferred onto an Immobilon-PVDF membrane and immunoblotted with an anti-phosphotyrosine antibody (Calbiochem, Billerica, MA). The membrane was stripped and re-blotted with anti-β-actin to ensure that the protein loading was equal among different samples.

For better resolution of the tyrosine-phosphorylated proteins, two-dimensional gel electrophoreses was performed on total urothelial proteins of the *Upk2*-HRAS^*/*^ transgenic mice (5 months of age) that harbored low-grade papillary UC. Briefly, the proteins were first resolved on an isoelectrofocusing gel containing ampholytes ranging from pH 4-8 and then on a 15% SDS gel. Two sets of the SDS gels were run under identical conditions, one stained with Coomassie Blue and stored at 4°C, and another transferred immediately onto an Immobilon-PVDF membrane and blotted with anti-phosphotyrosine antibody. The reactive spots on the X-ray film from the immunoblotting were matched with those on the Coomassie blue-stained gel, and the matching spots were excised and subjected to in-gel trypsin digestion. After the tryptic peptides were electroeluted from the gel, they were separated by reverse-phase HPLC and the individual peptides were subject to MALDI-mass spectrometry for sequence identification.

### Human urothelial carcinoma

Normal urothelial tissues were obtained from the bladder neck regions of patients who underwent radical prostatectomy (n=6); low-grade UC from patients who underwent transurethral resection of UC (n=10) and high-grade UC from patients who underwent radical cystectomy (n=10), following a protocol approved by the institutional review board of New York University School of Medicine. De-identified fresh tissues were subjected to either immediate protein extraction in SDS-PAGE loading buffer (see above for mouse tissues) for Western blotting or fixation, paraffin-embedding and sectioning for immunohistochemistry (see below).

### Culture of human primary and immortalized urothelial cells and urothelial carcinoma cell lines

Primary cultured normal human urothelial cells were purchased from ScienCell (Carlsbad, CA) and passaged 2-3 times in urothelial cell medium and growth supplement in the presence of 1% penicillin/streptomycin, all of which were provided by the manufacturer. UROtsa, an SV40T-immortalized urothelial cell line, was a kind gift from Dr. Ricardo Saban of University of Oklahoma Healthscience Center and were grown in DMEM/Ham's F12 (1:1) supplemented with 10% fetal bovine serum. T24T was a kind gift of Dr. Dan Theodorescu of University of Colorado. All other cell lines were purchased from ATCC and cultivated in the presence of 10% fetal bovine serum and the following media: RT4 in McCoy's 5A, RT112 in RPMI 1640, and J82, T24 and UMUC3 in DMEM. All the commercial cell lines were used within 6 months after their receipt. Authentication of the cell lines at ATCC was done by short tandem repeat profiling and isoenzyme analysis.

### siRNA knockdown of PKM2, PKM1 or both

Human UC cell lines T24T and RT4 were cultured (see above) to 70% confluence and transfected using lipofectamine 2000 (Invitrogen) with siRNAs specific for PKM1 (corresponding to exon 9 sequence of the human PKM gene), PKM2 (corresponding to exon 10 of the human PKM gene) or PKM (corresponding to regions common to PKM1 and PKM2). The designations, siRNA sequences and their efficiency ranking scores (based on Web-based Invitrogen siRNA design software) were: (1) for PKM1, PKM1-a: 5’-GCGUGGAGGCUUCUUAUAAdTdT-3’ (4 stars); PKM1-b: 5’-CGUGGAGGCUUCUUAUAAGdTdT-3’ (3.5 stars); PKM1-c: 5’-GAGGCUUCUUAUAAGUGUU dTdT-3’ (2.5 stars)); (2) for PKM2, PKM2-a: 5’-CCAUAAUCGUCCUCACCAA dTdT-3’ (4 stars); PKM2-b: 5’-AGGCAGAGGCUGCCAUCUA dTdT-3’ (3.5 stars); PKM2-c: 5’-GCCAUAAUCGUCCUCACCA dTdT-3’ (2.5 stars); (3) for PKM, PKM-T-a: 5’-GCUGUGGCUCUAGACACUAdTdT-3’ (5 stars); and PKM-T-b: 5’-GGACCUGAGAUCCGAACUGdTdT-3’ (5 stars). siRNA specific for EGFP (5’-CUUACGCUGAGUACUUCGA dTdT-3’) was used as a negative control. The specificity of the siRNAs and the extent of target knockdown were determined by RT-PCR using primers specific for PKM2 or PKM1. Twenty-hours after transfection, the cells were collected and subject to WST-1 cell proliferation assay (Sigma-Aldrich, St. Louis, MO).

### shRNA knockdown of PKM2 and assessment of cellular responses

A lentiviral approach was used that involved a three-plasmid system: (i) transfer plasmids (e.g., pLKO.1-TRC control, pLKO.1-TRC shPKM1 and pLKO.1-TRC shPKM2), (ii) packaging plasmid psPAX2 and (iii) envelope plasmid pMD2.G (all plasmids from Addgene, Cambridge, MA). The viruses were packaged in HEK 293T cells and used to transduce bladder cancer cell line T24 in the presence of 8 μg/mL polybrene. The shRNA for PKM1 was (sense: CCGGCAGCGTGGAGGCTTCTTATAACTCGAGTTATAAGAAGCCTCCACGCTGTTTTTG; anti-sense: AATTCAAAAACAGCGTGGAGGCTTCTTATAACTCGAGTTATAAGAAGCCTCCACGCTG). The shRNA for PKM2 was (sense: CCGGAGGCAGAGGCTGCCATCTACCCTCGAGGGTAGATGGCAGCCTCTGCCTTTTTTG; anti-sense: AATTCAAAAAAGGCAGAGGCTGCCATCTACCCTCGAGGGTAGATGGCAGCCTCTGCCT). Infected T24 cells were sub-cultured in a hypoxia chamber (Billups-Rothenberg Inc, Del Mar, CA) with 5% CO_2_ and 1% O_2_. After 36 hours of incubation, the cells were subject to Western blotting using antibodies against (i) PKM1 and PKM2; (ii) apoptotic markers cleaved caspase 3 and cleaved caspase 7; (iii) autophagy markers Atg 7 and LC3-I/II; and marker for unfolded protein response IRE1. All the primary antibodies were purchased from Cell Signaling Technology (Danvers, MA), except that anti-PKM1 was purchased from Sigma-Aldrich (St. Louis, MO). The level of apoptosis and cell-cycle distribution were assessed using an Annexin V-FITC apoptosis staining kit and a propidium iodide staining kit, respectively (both from Cell Signaling (Danvers, MA), using fluorescence-activated cell sorting. All samples were analyzed in triplicate.

### Histochemistry, immunofluorescence and immunohistochemistry and scoring and immunoprecipitation

Five micro-meter thick sections were cut from paraffin-embedded tissue blocks, deparaffinized and stained routinely with hematoxylin and eosin for histological examination. For antibody staining, the paraffin sections underwent antigen retrieval by microwave at maximum powder for 20 minutes in citrate buffer (pH 6.0). The sections were then stained consecutively with primary and secondary antibodies (fluorescein-conjugated for immunofluorescence and peroxidase-conjugated for immunohistochemistry). The antibodies and their dilutions were: anti-PKM2 (Cell Signaling Technology; 1:500), anti-phosphotyrosine (Cell Signaling Technology, Danvers; 1:200), anti-phosphorylated PKM2 (Cell Signaling Technology, Danvers, 1:200), anti-PKM1 (Sigma-Aldrich; 1:200), anti-cytokeratin 5 (Abcam; 1:500), and anti-cytokeratin 14 (Santa Cruz Biotechnology; 1:100). For scoring of immunohistochemical staining of human specimens, both the proportion and the intensity of the positive staining were scored following published methods [33], with the proportion graded in six scales (0-5; i.e., 0, none; 1, <1/100; 2, 1/100 to 1/10; 3, 1/10 to 1/3; 4, 1/3 to 2/3; and 5, more than 2/3), and the intensity graded in four scales (0-3; 0, none; 1, weak; 2, intermediate; and 3, strong). The total score from 0 to 8 was computed by combining the proportion and the intensity scores.

For immunofluorescence staining of cultured T24 cells, the cells were pre-fixed in 4% PBS-buffered paraformaldehyde and post-fixed and permeabilized in pre-cooled methanol/acetone mixture (1:1 ratio). The rest of the staining procedures were identical to those described for tissue sections.

For immunoprecipitation, total urothelial proteins dissolved in RIPA lysis and extraction buffer (Thermo Fisher Scientific Inc., Waltham, MA) containing a cocktail of protease and phosphatase inhibitors were incubated with anti-phosphotyrosine antibody. After further incubation with secondary antibody-conjugated resin, the resin was boiled in SDS. The eluent was resolved on a 15% SDS-PAGE, transferred to an Immobilon-PVDF membrane and immunoblotted with the anti-PKM2 antibody.

### Statistical analysis

Student's *t* test (two-tailed) was used to calculate the statistical significances with a *P* value <0.05 considered significant.

## References

[1] Alcolea MP, Jones PH (2013). Tracking cells in their native habitat: lineage tracing in epithelial neoplasia. Nat Rev Cancer.

[2] Wu XR (2005). Urothelial tumorigenesis: a tale of divergent pathways. Nat Rev Cancer.

[3] Wu XR (2009). Biology of urothelial tumorigenesis: insights from genetically engineered mice. Cancer Metastasis Rev.

[4] Knowles MA, Hurst CD (2015). Molecular biology of bladder cancer: new insights into pathogenesis and clinical diversity. Nat Rev Cancer.

[5] Dinney CP, McConkey DJ, Millikan RE, Wu X, Bar-Eli M, Adam L, Kamat AM, Siefker-Radtke AO, Tuziak T, Sabichi AL, Grossman HB, Benedict WF, Czerniak B (2004). Focus on bladder cancer. Cancer Cell.

[6] Castillo-Martin M, Domingo-Domenech J, Karni-Schmidt O, Matos T, Cordon-Cardo C (2010). Molecular pathways of urothelial development and bladder tumorigenesis. Urol Oncol.

[7] Hartmann A, Moser K, Kriegmair M, Hofstetter A, Hofstaedter F, Knuechel R (1999). Frequent genetic alterations in simple urothelial hyperplasias of the bladder in patients with papillary urothelial carcinoma. Am J Pathol.

[8] Choi W, Czerniak B, Ochoa A, Su X, Siefker-Radtke A, Dinney C, McConkey DJ (2014). Intrinsic basal and luminal subtypes of muscle-invasive bladder cancer. Nat Rev Urol.

[9] Zhang ZT, Pak J, Huang HY, Shapiro E, Sun TT, Pellicer A, Wu XR (2001). Role of Ha-ras activation in superficial papillary pathway of urothelial tumor formation. Oncogene.

[10] Mo L, Zheng X, Huang HY, Shapiro E, Lepor H, Cordon-Cardo C, Sun TT, Wu XR (2007). Hyperactivation of Ha-ras oncogene, but not Ink4a/Arf deficiency, triggers bladder tumorigenesis. J Clin Invest.

[11] Bayley JP, Devilee P (2012). The Warburg effect in 2012. Curr Opin Oncol.

[12] Mazurek S (2011). Pyruvate kinase type M2: a key regulator of the metabolic budget system in tumor cells. Int J Biochem Cell Biol.

[13] Gupta V, Bamezai RN (2010). Human pyruvate kinase M2: a multifunctional protein. Protein Sci.

[14] Lu Z (2012). Nonmetabolic functions of pyruvate kinase isoform M2 in controlling cell cycle progression and tumorigenesis. Chin J Cancer.

[15] Chen M, Zhang J, Manley JL (2010). Turning on a fuel switch of cancer: hnRNP proteins regulate alternative splicing of pyruvate kinase mRNA. Cancer Res.

[16] Chaneton B, Hillmann P, Zheng L, Martin AC, Maddocks OD, Chokkathukalam A, Coyle JE, Jankevics A, Holding FP, Vousden KH, Frezza C, O'Reilly M, Gottlieb E (2012). Serine is a natural ligand and allosteric activator of pyruvate kinase M2. Nature.

[17] Keller KE, Tan IS, Lee YS (2012). SAICAR stimulates pyruvate kinase isoform M2 and promotes cancer cell survival in glucose-limited conditions. Science.

[18] He F, Melamed J, Tang MS, Huang C, Wu XR (2015). Oncogenic HRAS Activates Epithelial-to-Mesenchymal Transition and Confers Stemness to p53-Deficient Urothelial Cells to Drive Muscle Invasion of Basal Subtype Carcinomas. Cancer Res.

[19] Van Batavia J, Yamany T, Molotkov A, Dan H, Mansukhani M, Batourina E, Schneider K, Oyon D, Dunlop M, Wu XR, Cordon-Cardo C, Mendelsohn C (2014). Bladder cancers arise from distinct urothelial sub-populations. Nat Cell Biol.

[20] Ho PL, Kurtova A, Chan KS (2012). Normal and neoplastic urothelial stem cells: getting to the root of the problem. Nat Rev Urol.

[21] DeGraff DJ, Robinson VL, Shah JB, Brandt WD, Sonpavde G, Kang Y, Liebert M, Wu XR, Taylor JA (2013). Current preclinical models for the advancement of translational bladder cancer research. Mol Cancer Ther.

[22] Wu XR, Mendelsohn C, DeGraff DJ (2015). Tumorigenicity of RTK/RAS in urothelium. Oncoscience.

[23] Ahmad I, Singh LB, Foth M, Morris CA, Taketo MM, Wu XR, Leung HY, Sansom OJ, Iwata T (2011). K-Ras and beta-catenin mutations cooperate with Fgfr3 mutations in mice to promote tumorigenesis in the skin and lung, but not in the bladder. Dis Model Mech.

[24] Chen X, Mitsutake N, LaPerle K, Akeno N, Zanzonico P, Longo VA, Mitsutake S, Kimura ET, Geiger H, Santos E, Wendel HG, Franco A, Knauf JA, Fagin JA (2009). Endogenous expression of Hras(G12V) induces developmental defects and neoplasms with copy number imbalances of the oncogene. Proc Natl Acad Sci USA.

[25] Tsuruta H, Kishimoto H, Sasaki T, Horie Y, Natsui M, Shibata Y, Hamada K, Yajima N, Kawahara K, Sasaki M, Tsuchiya N, Enomoto K, Mak TW, Nakano T, Habuchi T, Suzuki A (2006). Hyperplasia and carcinomas in Pten-deficient mice and reduced PTEN protein in human bladder cancer patients. Cancer Res.

[26] Yoo LI, Liu DW, Le Vu S, Bronson RT, Wu H, Yuan J (2006). Pten deficiency activates distinct downstream signaling pathways in a tissue-specific manner. Cancer Res.

[27] Sun Q, Chen X, Ma J, Peng H, Wang F, Zha X, Wang Y, Jing Y, Yang H, Chen R, Chang L, Zhang Y, Goto J, Onda H, Chen T, Wang MR (2011). Mammalian target of rapamycin up-regulation of pyruvate kinase isoenzyme type M2 is critical for aerobic glycolysis and tumor growth. Proc Natl Acad Sci USA.

[28] Yang W, Xia Y, Ji H, Zheng Y, Liang J, Huang W, Gao X, Aldape K, Lu Z (2011). Nuclear PKM2 regulates beta-catenin transactivation upon EGFR activation. Nature.

[29] Shirodkar SP, Lokeshwar VB (2009). Potential new urinary markers in the early detection of bladder cancer. Curr Opin Urol.

[30] Stadler WM, Lerner SP, Groshen S, Stein JP, Shi SR, Raghavan D, Esrig D, Steinberg G, Wood D, Klotz L, Hall C, Skinner DG, Cote RJ (2011). Phase III study of molecularly targeted adjuvant therapy in locally advanced urothelial cancer of the bladder based on p53 status. J Clin Oncol.

[31] Xylinas E, Kluth LA, Lotan Y, Daneshmand S, Rieken M, Karakiewicz PI, Shariat SF (2014). Blood- and tissue-based biomarkers for prediction of outcomes in urothelial carcinoma of the bladder. Urol Oncol.

[32] Galluzzi L, Senovilla L, Vitale I, Michels J, Martins I, Kepp O, Castedo M, Kroemer G (2012). Molecular mechanisms of cisplatin resistance. Oncogene.

[33] Harvey JM, Clark GM, Osborne CK, Allred DC (1999). Estrogen receptor status by immunohistochemistry is superior to the ligand-binding assay for predicting response to adjuvant endocrine therapy in breast cancer. J Clin Oncol.

